# Prevalence and correlates of smoking among urban adult men in Bangladesh: slum versus non-slum comparison

**DOI:** 10.1186/1471-2458-9-149

**Published:** 2009-05-22

**Authors:** Md Mobarak Hossain Khan, Aklimunnessa Khan, Alexander Kraemer, Mitsuru Mori

**Affiliations:** 1 Department of Public Health Medicine, School of Public Health, Bielefeld University, Germany; 2 Department of Public Health, Sapporo Medical University School of Medicine, Japan

## Abstract

**Background:**

Smoking is one of the leading causes of premature death particularly in developing countries. The prevalence of smoking is high among the general male population in Bangladesh. Unfortunately smoking information including correlates of smoking in the cities especially in the urban slums is very scarce, although urbanization is rapid in Bangladesh and slums are growing quickly in its major cities. Therefore this study reported prevalences of cigarette and *bidi *smoking and their correlates separately by urban slums and non-slums in Bangladesh.

**Methods:**

We used secondary data which was collected by the 2006 Urban Health Survey. The data were representative for the urban areas in Bangladesh. Both slums and non-slums located in the six City Corporations were considered. Slums in the cities were identified by two steps, first by using the satellite images and secondly by ground truthing. At the next stage, several clusters of households were selected by using proportional sampling. Then from each of the selected clusters, about 25 households were randomly selected. Information of a total of 12,155 adult men, aged 15–59 years, was analyzed by stratifying them into slum (= 6,488) and non-slum (= 5,667) groups. Simple frequency, bivariable and multivariable logistic regression analyses were performed using SPSS.

**Results:**

Overall smoking prevalence for the total sample was 53.6% with significantly higher prevalences among men in slums (59.8%) than non-slums (46.4%). Respondents living in slums reported a significantly (P < 0.001) higher prevalence of smoking cigarettes (53.3%) as compared to those living in non-slums (44.6%). A similar pattern was found for *bidis *(slums = 11.4% and non-slums = 3.2%, P < 0.001). Multivariable logistic regression revealed significantly higher odds ratio (OR) of smoking cigarettes (OR = 1.12, 95% CI = 1.03–1.22), *bidis *(OR = 1.90, 95% CI = 1.58–2.29) and any of the two (OR = 1.23, 95% CI = 1.13–1.34) among men living in slums as compared to those living in non-slums when controlled for age, division, education, marital status, religion, birth place and types of work. Division, education and types of work were the common significant correlates for both cigarette and *bidi *smoking in slums and non-slums by multivariable logistic regressions. Other significant correlates of smoking cigarettes were marital status (both areas), birth place (slums), and religion (non-slums). Similarly significant factors for smoking *bidis *were age (both areas), marital status (slums), religion (non-slums), and birth place (both areas).

**Conclusion:**

The men living in the urban slums reported higher rates of smoking cigarettes and *bidis *as compared to men living in the urban non-slums. Some of the significant correlates of smoking e.g. education and division should be considered for prevention activities. Our findings clearly underscore the necessity of interventions and preventions by policy makers, public health experts and other stakeholders in slums because smoking was more prevalent in the slum communities with detrimental health sequelae.

## Background

Smoking is a global public health concern. About 1.1 billion people smoke worldwide, which is expected to rise to more than 1.6 billion by 2025 [1]. It causes huge premature deaths [1-3] and poses considerable economic burden among the poor people especially living in developing countries like Bangladesh [4]. Worldwide the toll of tobacco is already high (Gavin, 2004). Unfortunately the low and middle income countries will experience more tobacco-attributable deaths in future decades [5-7]. Worldwide tobacco-attributable deaths were 4.83 million in 2000 [6], which are projected to reach at 6.4 million in 2015 and 8.3 million in 2030. In the low- and middle income countries such deaths are projected to increase from 3.4 million to 6.8 million between 2002 and 2030 [5].

Smoking cigarettes and *bidis *are common habits among the general male population in Bangladesh [8-10]. Smoking related diseases such as pulmonary diseases, stroke, ischemic heart disease, lung cancer and oral cancer are well documented in literature [9,11-14]. In the regions where the tuberculosis is prevalent, smokers have greater risk of dying from pulmonary tuberculosis as compared to non-smokers [1]. Tobacco related illnesses accounted for 16% of the total deaths among the general population of Bangladesh who are aged 30 years and above [13]. Smoking is also positively linked with the illicit drug use in Bangladesh, which is another public health concern [9]. The cost of tobacco consumption at the national level is found to be associated with the increased health-care costs, loss of productivity due to illnesses and early deaths and environmental damages [13]. Excess mortalities from all causes and cause-specific diseases are also reported in some countries [15,16] including India [11,17,18].

Urbanization is a worldwide phenomenon mostly occurring in developing countries [19]. Over the last two decades, many urban areas have expanded dramatically due to the rapid population growth, rural-urban migration and continued global economic integration [20,21]. In the near future, the pace of urbanization will be even faster as compared to the past. Recent data shows that the urban population in the world will become 4.58 billion by 2025, which was 3.29 billion in 2007. In contrast, the rural population will become 3.43 billion by 2025 which was 3.38 billion in 2007 [19]. Thus, virtually all the population growth (over 96%) over the next two decades will occur in the urban areas. Further analyses of the data indicate that most of the urban growth will occur in the less developed regions (1.21 billion out of 1.29 billion) mostly in Asian cities [19]. Unfortunately worldwide urbanization along with a large proportion of the slum populations in the urban areas has posed a public health problem. Already more than one billion people live in the slum areas mostly in developing countries and experts have estimated that the slum population will become double by 2030 [22].

Like other developing countries, Bangladesh has also experienced the same phenomena in terms of urbanization and population growth in the slum areas. About 25% of the total population live in the urban areas of Bangladesh and a large portion of the urban population live in slums mainly in the six divisional cities, ranging from 19.5% in Khulna City Corporation to 37.4% in the Dhaka megacity [23]. For instance, the slum population in the city of Dhaka increased sharply from 20% in 1996 to 37% in 2005 mainly due to rapid rural-urban migration [22,23]. About 300,000 to 400,000 new migrants are coming annually to the city of Dhaka, and most of them are trying to settle in the slum areas [24]. The slum areas differ from the non-slum areas by various ways. In the slum areas, poverty, overcrowding, poor housing, informal economical activities, lack of infrastructures, poor environmental and health facilities, and poor quality of life are generally common [22]. Crime, violence, and risky lifestyles such as smoking and use of illicit drugs are also commonly reported in the slum areas [24].

To our knowledge, no study explicitly analyzed smoking cigarettes and *bidis *including correlates of smoking among the general male population living in slums and non-slums located in the six major divisional cities of Bangladesh. Therefore, such information is seriously lacking in case of Bangladesh. Moreover, identification of smoking correlates is important to reduce the prevalence of smoking and associated consequences by developing suitable prevention policies. Particularly such a study is important in Bangladesh where the growth of slum populations and urbanization are really rapid. It is also important because higher smoking rates are reported in the overcrowded areas like slums [25-28]. Considering the above-mentioned background, this study reported the smoking prevalences including some of the socio-demographic correlates by slum and non-slum areas in the urban areas of Bangladesh. This study used the representative data of the urban areas of Bangladesh which were collected by the 2006 Urban Health Survey (UHS).

## Methods

### Study areas

Detailed information about the study design and areas of the UHS is given elsewhere [29]. Briefly, the UHS 2006 is a representative sample survey for the urban areas of Bangladesh. The main focus of the survey was the slum and non-slum areas of the six City Corporations (also called divisional cities) namely Barisal, Chittagong, Dhaka, Khulna, Rajshahi, and Sylhet. The 2006 UHS and the mapping of the urban slums were coordinated by the National Institute of Population Research and Training (NIPORT), Bangladesh. To prepare the maps of the urban slums in the above-mentioned cities, following activities were conducted. At the first phase, the satellite images were used to prepare the baseline maps of the City Corporations. The images were also used to identify the suspected slum settlements in the cities. To do this, the visual assessments of the satellite images were performed by experts who mainly focused on the settlement density and building materials. The identified slums by this process became the basis for the second phase. The second phase is referred to as "ground truthing" phase. In this phase, expert teams traveled into the each ward of the city to assess the ground conditions. It was necessary to verify the suspected slums in the phase one or to identify missing/new slums which were not obvious from the satellite images. Five criteria namely poor housing conditions, high population density, poor environmental services, high prevalence of poverty, and insecurity of tenure were used to verify the urban slums. Field team members declared a settlement as slum if it met four of these five criteria. Mapping of the urban slums was done by the Centre for Urban Studies, Dhaka [23]. This survey was funded by the United States Agency for International Development (USAID)/Bangladesh.

The 2006 UHS was conducted by the NIPORT, which is a research and training organization of the Government of Bangladesh. Before conducting the survey, this organization took the approval from the Ministry of Health and Family Welfare, which means that the study was ethically approved. Besides, the Technical Review Committee (TRC) (consisted of experts from government, non-governmental and international organizations as well as researchers and professionals working in the Health Nutrition and Population Sectors) of the NIPORT was responsible to look into the ethical issues. A Technical Task Force was also formed with the representatives from NIPORT, ICDDR, B, USAID/Bangladesh, Centre for Urban Studies, and MEASURE Evaluation for designing and implementing the survey. A verbal consent was also obtained from each respondent by explaining the objectives of the survey. The consent form clearly described the purpose of the study, confidentiality of the interviews, and their rights to participate voluntarily and to withdraw from the study at any point in time without any consequences. It was also assured that the information will be kept confidential.

### Domains and slum versus non-slum areas

The 2006 UHS had eight domains of areas namely (i) Dhaka Metropolitan large slum areas (by population), (ii) Dhaka Metropolitan small/medium slum areas (by population), (iii) Dhaka Metropolitan non-slum areas, (iv) Chittagong City Corporation slum areas, (v) Chittagong City Corporation non-slum areas, (vi) Slum areas of the remaining (Khulna, Rajshahi, Barisal, and Sylhet) City Corporations, (vii) Non-slum areas of the remaining City Corporations, and (viii) District Municipalities. As no distinction was made between slum and non-slum areas within district municipalities, we excluded this particular domain from analyses. Thus we used the data of seven domains in this paper. However, for better understanding of the results we made two broad domains by combining four domains of the slum areas and three domains of the non-slum areas. From each domain, 64 primary sampling units (PSUs) (also called clusters of households) were selected randomly with probability of selection proportional to population size. Households within selected PSUs were listed thoroughly, which served as the secondary sampling unit. From this master list, about 25 households per PSU were selected randomly, typically starting from the Northeast corner of the PSU. The intended total sample of households was 12,800 (spread across 512 communities in eight domains), of which 12,069 households with a response rate of 94.7% were successfully interviewed by the trained interviewers.

### Target Population and sample size

Within selected households, the target population for interview was all adults (men and women irrespective of marital status) aged 18–59 years and all ever-married adults aged 10–17 years. The present study used only the information of 12,155 men who were living in the selected clusters of the six City Corporations. We excluded the information of 1,664 men because they were living in the clusters of district municipalities. The response rates were 88.6% (n = 6,488 from 6,022 households) and 85.1% (n = 5,667 from 4,522 households) in the urban slum and non-slum areas, respectively. The survey was conducted between February and July 2006.

### Data collection tools

The 2006 UHS was a multi-level study designed to illustrate circumstances at the community, household and individual level. Therefore four different questionnaires related to household, male, female and neighborhood were employed. All these questionnaires were pre-tested and modified accordingly (based on field experiences) before conducting final surveys. Since the present study was based on the male data, we provided some more information about the male questionnaire. This questionnaire included basic individual characteristics, employment history, migration history, health care decision making, general health, activities of daily living, injuries, knowledge of AIDS and other sexually transmitted diseases, mental health, domestic violence, smoking, drug use and crime. Before conducting the interview, the interviewer explained the objectives of the survey to the respondents for getting their consent.

### Data management and quality

The fieldwork for the 2006 UHS was conducted by the Associates for Community and Population Research (ACPR) based in Dhaka, Bangladesh. The whole survey was monitored and supervised by the experts of NIPORT and ICDDR, B to assure the quality of data. The data were processed on microcomputers by the staffs of ACPR, who were trained for data entry, editing and coding beforehand.

### Dependent variable

Following variables were used as dependent variables:

• Do you smoke cigarette currently?

• Do you smoke *bidi *currently?

Each respondent was asked to report either 'yes' or 'no' for each question. For descriptive analysis, we combined these variables to make another composite variable (called any of the two). When 'yes' was reported for any of the two variables, then the respondent was considered as 'smoker', otherwise as 'non-smoker'. Two more variables of smoking were also used for descriptive analysis:

• How many cigarettes do you smoke in a typical day?

• How many *bidis *do you smoke in a typical day?

We performed separate analyses because cigarette and *bidi *are different in terms of price and quality. Generally *bidis *are the cheapest substitutes of cigarettes and mostly consumed by the poor people. *Bidis *contain more tar and nicotine than cigarettes which are more risky for health [8,9,30].

### Independent variables

Independent variables namely age, division, education, marital status, religion, birth place, and types of work (i.e. derived from the question 'for whom do you wok?') including 'whether the respondent living in urban slum or not' were considered for the analysis.

### Analysis

Simple frequency, bivariable and binary multivariable logistic regression analyses were performed using SPSS. Frequency analysis was performed to show the prevalences of smoking cigarettes and *bidis *by the urban slum and non-slum areas. Bivariable and multivariable logistic regression analyses were mainly performed to identify the important correlates of the smoking variables by stratifying the male populations living in the urban slums and non-slum areas. The advantage of using binary multivariable logistic regression analysis is that it can include many independent variables at the same time and the findings are easy to interpret. In this study, results are mainly reported by the prevalences of smoking variables, by odds ratios (OR) and 95% confidence intervals (CI). P-values are also reported to show the significance level of the test.

## Results

**Figure 1:**
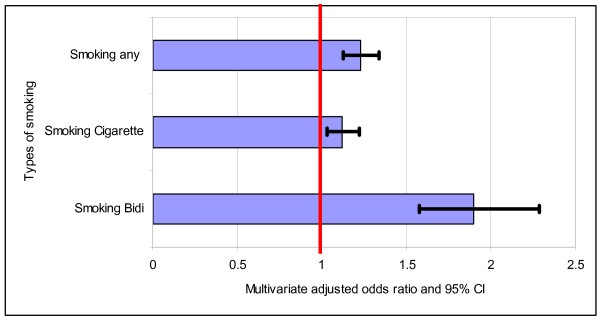
**Odds ratio of smoking including 95% confidence interval by slum versus non-slum men adjusted for age, division, education, marital status, religion, birth place and types of work**.

The prevalences of smoking cigarettes, *bidis *and any of the two (Table 1) for the total sample were 49.3%, 7.6% and 53.6%, respectively. The prevalences for both cigarette and *bidi *smoking were significantly higher among males living in the urban slums as compared to the males living in urban non-slums. For instance, the prevalence of *bidi *smoking was 11.4% and 3.2% among males in the urban slums and non-slums, respectively. The daily averages of smoking cigarettes and *bidis *(in number) among male smokers were 9.9 and 13.9 in slums as compared to 9.3 and 13.3 in non-slums, respectively. For total sample, the multivariable logistic regression analyses controlled for age, division, education, marital status, religion, birth place and types of work indicated significantly higher odds ratio of smoking cigarettes, *bidis *and any of the two among the male population in urban slums than those living in non-slums (Figure 1). For this reason, we analyzed correlates of smoking cigarettes and *bidis *separately by the urban slum and non-slum areas. It should be noted that all the controlled variables were significant in the multivariable logistic regression models except the variable 'birth place' for cigarette smoking and any of the two (not shown).

**Table 1:** Prevalence of smoking variables among men by the urban slum and non-slum areas in Bangladesh

Variables	Slum		Non-slum		Total		P
	
	n	%	n	%	n	%	
Currently smoke cigarette:							
No	3027	46.7	3138	55.4	6155	50.7	< 0.001
Yes	3461	53.3	2529	44.6	5990	49.3	
							
No. of cigarette on a typical day:							
No	3027	46.7	3138	55.4	6165	50.7	< 0.001
1–10	2594	40.0	1906	33.6	4500	37.0	
11–20	729	11.2	510	9.0	1239	10.2	
21+	138	2.1	113	2.0	251	2.1	
Average (daily)		9.9		9.3			
							
Currently smoke *bidi*:							
No	5748	88.6	5487	96.8	11235	92.4	< 0.001
Yes	740	11.4	180	3.2	920	7.6	
							
No. of *bidi *on a typical day:							
No	5748	88.6	5487	96.8	11235	92.4	< 0.001
1–10	371	5.7	78	1.4	449	3.7	
11–20	190	2.9	65	1.1	255	2.1	
21+	178	2.7	36	0.6	214	1.8	
Average (daily)		13.9		13.3			
							
Currently smoking any of the two:							
None	2606	40.2	3037	53.6	5643	46.4	< 0.001
Only cigarette	3142	48.4	2450	43.2	5592	46.0	
Only *bidi*	421	6.5	101	1.8	522	4.3	
Both	319	4.9	79	1.4	398	3.3	

Table 2 shows the prevalences of smoking cigarettes, *bidis *and any of the two (obtained from bivariate analyses) by some selected variables in the urban slum areas. Almost all the variables were significantly associated with smoking variables except religion for cigarette smoking and any of the two. For instance, the prevalence of smoking *bidis *was significantly higher among those men who were: older, living in Khulna and Sylhet divisions, uneducated, divorced/separated/widowed, Christian, born in district towns and villages, and day laborers.

**Table 2:** Prevalence of currently smoking (%) by background characteristics of men living in the urban slum areas

Variables	n	Cigarette (%)	*Bidi (%)*	Any of the two (%)
**Age:**				
15–29	2794	49.0	5.2	50.7
30–44	2280	58.6	14.0	66.7
45–59	1413	53.5	19.4	66.7
		P = 0.000	P = 0.000	P = 0.000
**Division:**				
Barisal	240	48.5	15.8	57.9
Chittagong	1617	52.4	8.3	58.0
Dhaka	3286	54.0	10.8	60.0
Khulna	537	42.3	20.1	51.4
Rajshahi	618	60.4	11.8	65.9
Sylhet	190	64.7	15.8	79.1
		P = 0.000	P = 0.000	P = 0.000
**Education:**				
No	2195	59.2	21.3	73.2
Primary	1881	55.4	10.2	59.8
Secondary	1941	49.4	4.1	51.0
Higher secondary	471	34.2	0.6	34.4
		P = 0.000	P = 0.000	P = 0.000
**Marital status:**				
Never married	1427	40.4	1.7	40.9
Married	5015	57.0	14.2	65.2
Divorced/S/W/D	46	53.2	15.2	63.0
		P = 0.000	P = 0.000	P = 0.000
**Religion:**				
Islam	6143	53.3	11.6	59.9
Hinduism	314	54.3	7.3	57.5
Buddhism	10	50.0	0.0	50.0
Christianity	21	57.1	23.8	71.4
		P = 0.962	P = 0.020	P = 0.512
**Birth place:**				
City corporation	1757	54.0	6.3	56.9
District town	600	49.1	14.3	56.4
Other town	149	60.4	8.7	64.4
Village	3925	53.5	13.4	61.5
Abroad	57	49.1	10.5	56.1
		P = 0.087	P = 0.000	P = 0.004
**Types of work:**				
Not working	364	39.4	5.2	43.7
For family business	109	39.4	3.6	41.3
For private company	1962	48.0	4.7	50.1
For Government	298	41.6	5.0	43.0
Self employed	1830	57.1	10.9	63.3
Day labor	1925	60.5	21.3	73.2
		P = 0.000	P = 0.000	P = 0.000

Table 3 reveals the results of bivariate analyses in non-slum areas. All the selected variables were significantly associated with all types of smoking. For instance, the prevalence of smoking cigarettes was lowest among those men who were: youngest (15–29 years), living in Barisal division, educated (11+ years education), never married, Islamic (means the religion was Islam), born in other (smaller) towns, and not working.

**Table 3:** Prevalence of current smoking (%) by background characteristics of men living in the urban non slum areas

Variables	n	Cigarette (%)	*Bidi (%)*	Any of the two (%)
**Age:**				
15–29	2431	38.7	0.3	38.8
30–44	2065	50.9	3.6	53.2
45–59	1171	45.7	8.5	50.3
		P = 0.000	P = 0.000	P = 0.000
**Division:**				
Barisal	203	34.5	6.5	38.6
Chittagong	2008	44.8	1.7	46.3
Dhaka	1846	44.4	2.3	45.4
Khulna	536	41.8	10.4	46.8
Rajshahi	494	50.4	5.3	52.3
Sylhet	580	46.4	1.4	47.6
		P = 0.003	P = 0.000	P = 0.023
**Education:**				
No	764	59.0	11.5	65.8
Primary	1003	47.3	5.6	50.9
Secondary	2113	45.6	1.7	46.1
Higher secondary	1787	35.9	0.1	36.0
		P = 0.000	P = 0.000	P = 0.000
**Marital status:**				
Never married	1950	34.4	0.2	34.5
Married	3676	49.8	4.8	52.5
Divorced/S/W/D	41	68.3	2.4	70.7
		P = 0.000	P = 0.000	P = 0.000
**Religion:**				
Islam	4730	44.1	3.4	46.2
Hinduism	832	45.3	1.3	45.9
Buddhism	32	59.4	0.0	59.4
Christianity	73	61.6	12.3	63.0
		P = 0.007	P = 0.000	P = 0.015
**Birth place:**				
City corporation	1641	42.0	1.3	42.4
District town	818	48.0	5.0	51.0
Other town	162	38.3	0.6	38.3
Village	2986	45.2	3.9	47.5
Abroad	60	60.0	1.6	60.0
		P = 0.002	P = 0.000	P = 0.000
**Types of work:**				
Not working	674	28.0	1.2	28.5
For family business	252	47.2	0.0	47.2
For private company	1849	44.7	1.1	44.7
For Government	584	44.5	1.4	45.0
Self employed	1663	47.3	2.8	48.8
Day labor	654	54.0	15.0	63.4
		P = 0.000	P = 0.000	P = 0.000

The results (ORs and 95% CIs) of binary multivariable logistic regression analyses for 'smoking cigarettes' are shown in Table 4. Age and religion lost their significance levels when analyses were restricted to the urban slums. All other variables remained significant in the multivariable logistic regression. Similarly, all the selected variables except age were significantly associated with smoking cigarettes in the urban non-slum areas. The male population who were from Rajshahi and Sylhet divisions, uneducated, married, self-employed and day laborer reported consistently higher odds of smoking cigarettes in both slum and non-slum areas. According to Table 5, smoking *bidis *was significantly associated with all the selected correlates except religion in the urban slum areas. Particularly the male population who were aged 30 and above, who had no education, who were born in district towns and villages, and who were day laborers reported consistently higher odds of smoking *bidis *in both slum and non-slum areas.

**Table 4:** Association of smoking cigarette by background characteristics of men living in the urban slum and non slum areas

	Slum		Non-slum	
Variables	OR (95% CI)	P	OR (95% CI)	P
**Age:**				
15–29	1.00		1.00	
30–44	1.10 (0.96–1.25)	0.183	1.13 (0.96–1.32)	0.152
45–59	0.90 (0.77–1.04)	0.158	0.86 (0.71–1.04)	0.110
**Division:**				
Barisal	1.00		1.00	
Chittagong	1.33 (1.01–1.76)	0.046	1.43 (1.04–1.96)	0.027
Dhaka	1.41 (1.08–1.85)	0.012	1.42 (1.03–1.95)	0.030
Khulna	0.85 (0.62–1.17)	0.309	1.14 (0.80–1.63)	0.460
Rajshahi	1.73 (1.27–2.37)	0.001	1.96 (1.38–2.80)	0.000
Sylhet	1.95 (1.30–2.90)	0.001	1.53 (1.07–2.18)	0.019
**Education:**				
No	1.00		1.00	
Primary	0.99 (0.87–1.13)	0.866	0.68 (0.56–0.83)	0.000
Secondary	0.86 (0.75–0.99)	0.030	0.71 (0.59–0.85)	0.000
Higher secondary	0.49 (0.39–0.62)	0.000	0.49 (0.40–0.59)	0.000
**Marital status:**				
Never married	1.00		1.00	
Married	1.73 (1.49–2.02)	0.000	1.56 (1.32–1.85)	0.000
Divorced/S/W/D	1.57 (0.86–2.89)	0.142	3.23 (1.65–6.31)	0.001
**Religion:**				
Islam	1.00		1.00	
Hinduism	1.14 (0.90–1.44)	0.297	1.11 (0.95–1.31)	0.179
Buddhism	1.47 (0.41–5.24)	0.554	1.95 (0.94–4.03)	0.071
Christianity	1.86 (0.76–4.55)	0.176	2.27 (1.39–3.70)	0.001
**Birth place:**				
City corporation	1.00		1.00	
District town	0.78 (0.64–0.95)	0.014	1.28 (1.06–1.53)	0.10
Other town	1.33 (0.93–1.90)	0.115	0.91 (0.64–1.28)	0.577
Village	0.85 (0.75–0.97)	0.017	1.05 (0.92–1.20)	0.452
Abroad	0.62 (0.36–1.06)	0.082	1.43 (0.83–2.47)	0.204
**Types of work:**				
Not working	1.00		1.00	
For family business	0.93 (0.60–1.46)	0.758	1.90 (1.39–2.58)	0.000
For private company	1.17 (0.93–1.49)	0.185	1.50 (1.22–1.84)	0.000
For Government	0.89 (0.64–1.23)	0.488	1.51 (1.17–1.96)	0.002
Self employed	1.51 (1.19–1.92)	0.001	1.63 (1.32–2.01)	0.000
Day labor	1.62 (1.26–2.06)	0.000	1.75 (1.35–2.26)	0.000

**Table 5:** Association of smoking *bidis *by background characteristics of men living in the urban slum and non slum areas

Variables:	Slum		Non-slum	
	OR (95% CI)	P	OR (95% CI)	P
**Age:**				
15–29	1.00		1.00	
30–44	1.58 (1.25–1.98)	0.000	6.82 (2.79–16.69)	0.000
45–59	2.35 (1.84–2.99)	0.000	18.89 (7.66–46.63)	0.000
**Division:**				
Barisal	1.00		1.00	
Chittagong	0.48 (0.31–0.72)	0.001	0.11 (0.05–0.26)	0.000
Dhaka	0.63 (0.43–0.94)	0.023	0.13 (0.06–0.29)	0.000
Khulna	1.34 (0.86–2.10)	0.195	0.53 (0.23–1.22)	0.133
Rajshahi	1.00 (0.63–1.60)	0.994	0.67 (0.28–1.60)	0.364
Sylhet	0.68 (0.39–1.18)	0.170	0.06 (0.02–0.19)	0.000
**Education:**				
No	1.00		1.00	
Primary	0.55 (0.45–0.66)	0.000	0.65 (0.43–0.98)	0.039
Secondary	0.29 (0.22–0.37)	0.000	0.28 (0.18–0.45)	0.000
Higher secondary	0.06 (0.02–0.19)	0.000	0.01 (0.00–0.08)	0.000
**Marital status:**				
Never married	1.00		1.00	
Married	3.22 (2.05–5.05)	0.000	1.71 (0.54–5.43)	0.367
Divorced/S/W/D	3.10 (1.18–8.15)	0.022	0.68 (0.08–5.71)	0.721
**Religion:**				
Islam	1.00		1.00	
Hinduism	0.86 (0.54–1.37)	0.530	0.59 (0.30–1.18)	0.135
Buddhism	-	-	-	-
Christianity	1.98 (0.64–6.09)	0.233	3.34 (1.37–8.13)	0.008
**Birth place:**				
City corporation	1.00		1.00	
District town	1.84 (1.31–2.57)	0.000	3.89 (2.02–7.50)	0.000
Other town	1.57 (0.82–3.01)	0.176	1.25 (0.12–12.68)	0.849
Village	1.80 (1.39–2.32)	0.000	4.47 (2.50–7.96)	0.000
Abroad	0.62 (0.24–1.57)	0.309	0.15 (0.01–1.50)	0.105
**Types of work:**				
Not working	1.00		1.00	
For family business	0.51 (0.15–1.67)	0.264	0.08 (0.00–3.25)	0.178
For private company	0.87 (0.51–1.49)	0.619	0.40 (0.16–1.01)	0.051
For Government	0.70 (0.34–1.45)	0.339	0.18 (0.06–0.58)	0.004
Self employed	1.27 (0.76–2.11)	0.364	0.55 (0.23–1.31)	0.177
Day labor	2.47 (1.49–4.09)	0.000	2.39 (1.02–5.58)	0.045

## Discussion

Our findings demonstrate that men living in the urban slum areas are more likely to smoke both cigarettes and *bidis *than their counterparts living in the urban non-slum areas even after controlling for many variables (Figure 1). In Bangladesh, the poorest people are living in slums in the urban areas, and they are characterized by many negative factors such as higher prevalences of poverty, lack of education, informal economical activities, unskilled professions, poor households and poor environmental conditions [22]. Although our results are not directly comparable because of limited information in the urban areas, these results are consistent with the findings of other studies in Bangladesh [4,8,9]. For instance, a study based on rural areas in Bangladesh reported that tobacco consumption was 2+ times more among the poor individuals (75.5%) than the rich (36.0%) [8]. Similarly, Efroymson et al [4] reported that the poorest households were 2 times more likely to smoke tobacco as compared to the wealthiest households in Bangladesh.

Other studies also reported higher smoking rates in deprived or overcrowded areas [25-28]. Unhealthy lifestyles in adverse socio-economic conditions (e.g. due to less education), social norms, cultural beliefs [26], neighborhood characters [31], poor environment, availability of cigarettes, and worse provision of preventive services in the deprived areas may have significant impact on smoking behaviors of individuals [27]. The detrimental effects of smoking on family members (e.g. children) who are especially living in the overcrowded areas are also reported by Irvine et al [32]. According to them, crowding in the home was positively associated with a higher cotinine (nicotine) level among children with asthma [32].

Although tobacco consumption is harmful for both users and their families, unfortunately the devastating effects could be more for those families who are living under poverty [33]. Even a small expenditure on tobacco consumption can enhance the poverty level of the poor families, interrupt food supplies, reduce health seeking behaviors, and increase prevalence of the malnutrition among the family members [8,30,33,34]. It should be mentioned that about 10 million malnourished people in Bangladesh could get adequate nutrition if they spend money on foods rather than spending on tobacco [4]. Inadequate health knowledge, limited health care facilities, and higher prevalence of malnutrition [22] could also aggravate the consequences of smoking among populations living in the urban slums. Our data indicated that the percentages of body mass index below 18.5 kg/m^2 ^were 34.9% and 19.7% among men living in the urban slum and non-slum areas, respectively. Among cigarette smokers, the prevalence of underweight was significantly higher in the urban slums as compared to the urban non-slums (slum = 39.6% and non-slum = 21.3%, P < 0.001). Such underweight prevalences were not significantly different among *bidi *smokers, (slum = 53.9% and non-slum = 47.8%). However, higher prevalences of smoking *bidis *in the urban areas should be a public health concern because *bidis *are more risky than cigarettes due to higher concentrations of tar and nicotine [9,30]. A positive association between tobacco consumption and use of illicit drugs in Bangladesh also indicates a public health concern [9]. In short, the male populations especially the smokers who are living in the urban slums need adequate public health attentions and preventions. It is also important because the populations living in the urban slums are increasing rapidly in Bangladesh [22,23].

Present study identified various significant factors which may be useful for developing some interventions in the urban areas. Smoking tobacco especially *bidis *was positively associated with age of men in the urban areas. Although this finding is not directly comparable with the finding of other studies mainly because of different study areas, but several studies reported such a positive association in other areas (not slums) of Bangladesh [9] and elsewhere [35-37]. According to the results of multivariable logistic regression models, the prevalences of smoking cigarettes (Table 4) and *bidis *(Table 5) were significantly different by divisions. For instance, odds of smoking cigarettes was significantly higher in both the urban slum and non-slum areas of Sylhet division, whereas smoking *bidis *was significantly lower in the urban non-slums of the same division. Similarly, Barisal division showed lower odds of smoking cigarettes but higher odds of smoking *bidis*. Unfortunately possible reasons of such differences by divisional cities were not explored by previous studies. Therefore, further studies are recommended in this regard.

Our study revealed an inverse association between education and smoking. Although such a negative association between *bidi *smoking and education was consistently reported by many other studies [9,38,39], the association between cigarette smoking and education was not consistently reported. Therefore further studies are recommended to investigate the reasons of such inconsistencies. Proper strategies are extremely needed in Bangladesh to increase the rate of education especially in the urban slum areas. Because more than 80% of the urban slum populations in Dhaka terminated their education before going to the secondary school [40]. The combined efforts by government, policy makers and other concerned bodies can improve the situation. According to our finding, men who were born in villages but now living in the urban slums (could be considered as rural migrants) reported significantly higher likelihood of smoking *bidis *as compared to those who were born in the City Corporation and now living in slums. Higher prevalences of smoking *bidis *in the rural areas as compared to the urban areas [9] might be another explanation for this. Probably the poor economic conditions of the migrants also influence them to smoke *bidis *which are the cheaper than cigarettes. Normally *bidi *smokers have less education and low income as compared to cigarette smokers [9,41,42]. Similar explanations may be applicable for the widowed/divorced men in Bangladesh. Unfortunately it is beyond our scope to discuss higher likelihoods of smoking cigarettes and *bidis *by Christians as compared to Muslims because of limited information. The significantly higher likelihood of smoking among day labors as compared to 'not working group' might be related with their low socio-economic status and deprivation, which were reported also by some other studies [26,27,31].

Tobacco-attributable deaths and diseases are largely preventable [43]. Unfortunately smoking is hardly perceived as a health hazard in Bangladesh [8]. Actions like (i) mass health education programs, (ii) ban of tobacco advertising and promotion, (iii) explicit health warnings on tobacco products, (iv) policies on taxation and restrictions on smoking in workplaces and in public places, and (v) diversification of crops in the countries with growing tobacco productions may be useful to reduce the consequences of tobacco consumption [2]. Some of these actions are already enacted in Bangladesh. For instance, advertisements of cigarettes or *bidi*s now include a warning message stating that smoking is harmful to health. Health warnings are also mandatory on packages of cigarettes and *bidi*s. Unfortunately these printed messages are not so effective in Bangladesh because about 50% of the population is still illiterate and hence they can not read the messages on packets. Moreover, many smokers buy single stick rather than the full packet of cigarettes. Therefore, they miss the warning message written on the packets [8]. Some more strategies such as involvement of religious leaders, health services providers [9], teachers, community leaders, and mass media can reduce tobacco consumption among males in the urban areas. As poor smokers are generally more price sensitive, increasing prices of tobacco might be another option to discourage them (e.g. who live in slums) to smoke. Save money by reducing or quitting smoking can also contribute to the family. Because in this case more money can be allocated for foods, other goods, and services. As a result, the nutritional status and health of the household members (like children) can be improved [44].

Some strengths of the study should be mentioned. A large sample of adult males from all the six City Corporations in Bangladesh was considered for the survey. Hence this study is representative for the urban areas in Bangladesh. Moreover, this is the first study, to our knowledge, which identified some correlates of smoking cigarettes and *bidis *by the urban slums and non-slums. A cross-sectional design of the survey was the main limitation which precluded us to comment on the cause-effect relationships of the significant associations.

## Conclusion

Likelihoods of smoking cigarettes and *bidis *were significantly higher among men in slums as compared to men in non-slums of the urban areas. As the poor people living in slums are more likely to smoke and they are at higher risk of smoking consequences, more interventions should be needed for them. All the significant variables should be considered for developing suitable policies to reduce the consequences of smoking in Bangladesh.

## Competing interests

The authors declare that they have no competing interests.

## Authors' contributions

MMHK framed the research question and performed statistical analyses. AK made the literature review and drafted the manuscript. AK and MM checked the manuscript, provided comments on the manuscript and contributed to the discussion section. All authors read and approved the final manuscript.

## Pre-publication history

The pre-publication history for this paper can be accessed here:


